# Incident venous thromboembolic events in the Prospective Study of Pravastatin in the Elderly at Risk (PROSPER)

**DOI:** 10.1186/1471-2318-11-8

**Published:** 2011-02-22

**Authors:** Dilys J Freeman, Michele Robertson, E Ann Brown, Ann Rumley, Edward S Tobias, Marijke Frölich, P Eline Slagboom, J Wouter Jukema, Anton JM de Craen, Naveed Sattar, Ian Ford, Allan Gaw, Ian A Greer, Gordon DO Lowe, David J Stott

**Affiliations:** 1 Centre of Population and Health Sciences, University of Glasgow, Glasgow, UK; 2 Robertson Centre for Biostatistics, University of Glasgow, Glasgow, UK; 3 Institute of Cardiovascular and Medical Sciences, Faculty of Medicine, University of Glasgow, Glasgow, UK; 4 Clinical Specialties, School of Medicine, University of Glasgow, Glasgow, UK; 5 Leiden University Medical Centre, Leiden, Netherlands; 6 Clinical Research Facility, University of Glasgow, Glasgow, UK

## Abstract

**Background:**

Venous thromboembolic events (VTE), including deep venous thrombosis and pulmonary embolism, are common in older age. It has been suggested that statins might reduce the risk of VTE however positive results from studies of middle aged subjects may not be generalisable to elderly people. We aimed to determine the effect of pravastatin on incident VTE in older people; we also studied the impact of clinical and plasma risk variables.

**Methods:**

This study was an analysis of incident VTE using data from the Prospective Study of Pravastatin in the Elderly at Risk (PROSPER), a randomized, double-blind, placebo-controlled trial of pravastatin in men and women aged 70-82. Mean follow-up was 3.2 years. Risk for VTE was examined in non-warfarin treated pravastatin (n = 2834) and placebo (n = 2865) patients using a Cox's proportional hazard model, and the impact of other risk factors assessed in a multivariate forward stepwise regression analysis. Baseline clinical characteristics, blood biochemistry and hematology variables, plasma levels of lipids and lipoproteins, and plasma markers of inflammation and adiposity were compared. Plasma markers of thrombosis and hemostasis were assessed in a nested case (n = 48) control (n = 93) study where the cohort was those participants, not on warfarin, for whom data were available.

**Results:**

There were 28 definite cases (1.0%) of incident VTE in the pravastatin group recipients and 20 cases (0.70%) in placebo recipients. Pravastatin did not reduce VTE in PROSPER compared to placebo [unadjusted hazard ratio (95% confidence interval) 1.42 (0.80, 2.52) p = 0.23]. Higher body mass index (BMI) [1.09 (1.02, 1.15) p = 0.0075], country [Scotland vs Netherlands 4.26 (1.00, 18.21) p = 0.050 and Ireland vs Netherlands 6.16 (1.46, 26.00) p = 0.013], lower systolic blood pressure [1.35 (1.03, 1.75) p = 0.027] and lower baseline Mini Mental State Examination (MMSE) score [1.19 (1.01, 1.41) p = 0.034] were associated with an increased risk of VTE, however only BMI, country and systolic blood pressure remained significant on multivariate analysis. In a nested case control study of definite VTE, plasma Factor VIII levels were associated with VTE [1.52 (1.01, 2.28), p = 0.044]. However no other measure of thrombosis and haemostasis was associated with increased risk of VTE.

**Conclusions:**

Pravastatin does not prevent VTE in elderly people at risk of vascular disease. Blood markers of haemostasis and inflammation are not strongly predictive of VTE in older age however BMI, country and lower systolic blood pressure are independently associated with VTE risk.

**Trial Registration:**

Not applicable when study undertaken.

## Background

Venous thromboembolism (VTE) has an incidence of 1-2 per 1000 individuals per year but is close to 1% per annum in those aged over 70 years[1]. VTE is a leading cause of death in hospital inpatients[2] and is a major cause of morbidity and mortality particularly in older people and among those with cancer[1,3]. Despite the fact that 70% of patients with VTE are aged over 60[1], there are few studies of risk factors in the elderly. Other studies have analyzed effects of pre-existing statin medication on incident VTE [4-6] or have compared statin use in case control studies of VTE[7-9]. A *post hoc *analysis of the Heart and Estrogen/progestin Replacement Study (HERS)[4] and analyses of other[5-8], but not all [9], population or case control studies indicated a decreased risk of VTE with statin use. A systematic review of observational studies suggested that statins may be useful in the prevention of VTE[10]. However, observational studies are prone to confounding hence randomized controlled trials are required to assess reliably the effects of statins on VTE risk.

Recently a randomized control trial of rosuvastatin in the prevention of VTE, in middle-aged subjects with low LDL cholesterol and raised C-reactive protein levels, (JUPITER)[11] indicated that rosuvastatin significantly reduced the occurrence of symptomatic VTE, (hazard ratio 0.57, 95% confidence interval 0.37 - 0.86, p = 0.007). Reviews of this trial and of recent case control studies [12,13] have renewed the debate on the efficacy of statins in the prevention of VTE and the call for analysis of prospective data. A meta-analysis suggested that statin treatment was likely to reduce the risk of VTE, however there was significant heterogeneity of study outcome [14], and as the majority of studies looked at middle-aged rather than elderly populations and there was no separate analysis by age, it was not certain that elderly people benefit.

The Prospective Study of Pravastatin in the Elderly at Risk (PROSPER) was a multi-centre, randomized, double-blind, placebo-controlled trial of pravastatin in the prevention of vascular disease in the elderly[15]. The present study is an analysis of incident VTE in this population of men and women aged 70-82 using data from the PROSPER database. The aim was to determine whether pravastatin reduces VTE incidence in older people. In addition, we assessed the impact of clinical, hematological, lipid, inflammatory and vascular risk factors for incident VTE in older age.

## Methods

### Subjects

**Figure 1:**
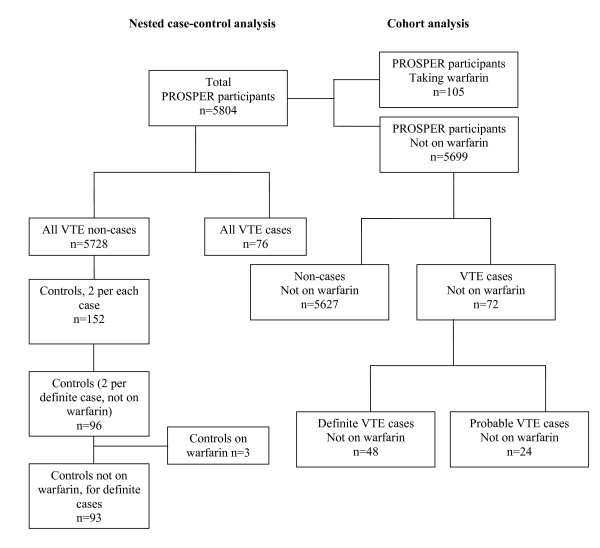
**Flow diagram demonstrating identification of VTE cases, non-cases and controls in PROSPER**.

The design and outcome of PROSPER is described elsewhere[15-17]. Men and women aged 70-82 (n = 5804) with evidence of pre-existing vascular disease or at least one major risk factor for vascular disease were randomized to placebo or pravastatin (40 mg per day) treatment. Follow up was 3.2 years on average. For the present study the full medical records were retrieved for all suspected VTE in the PROSPER database and examined by three clinicians in a pre-planned *post hoc *analysis. Events were categorized as definite VTE if death from VTE was confirmed on the death certificate or if there was recorded evidence from investigations such as ultrasound venography ventilation perfusion lung scans or computed tomography pulmonary angiography. Events were categorized as probable VTE when there was a record of at least 3 months' continuous anticoagulant treatment with warfarin or heparin or a clinician diagnosis of VTE in the trial records plus documentation of venous insufficiency or venous leg ulceration but no confirmation by clinical test or anticoagulant treatment. A consensus of two clinical opinions was required to confirm the event categorization. Where data were available for the entire cohort (Figure 1, Table 1), the utility of a parameter in predicting risk of VTE was assessed using all cases (n = 48 definite VTE, n = 72 combined definite and probable VTE) and non-cases (n = 5627) not on warfarin treatment. For hemostatic variables and IGF-1 (Table 2) a nested case control study was performed. We matched each probable and definite case (n = 76) with 2 controls (n = 152) selected at random from all non-cases on the basis of age (using two-year age categories), smoking status and country of origin (Figure 1). We excluded those controls that matched with a probable case, leaving 96 matching 48 definite cases, 3 of these 96 controls were on warfarin and removed from the analysis leaving 93 controls (Figure 1). In order to assess the impact of cancer as a precipitating factor for VTE, cases were separated into those who did and those who did not develop cancer during the study (diagnosis confirmed by endpoint committee). The institutional ethics review boards of all centres approved the protocol [the Argyll and Clyde Local Research Ethics Committee, the Glasgow Royal Infirmary Local Research Ethics Committee, Greater Glasgow Primary Care and Mental Health Research Ethics Committee, Lanarkshire Health Board Local Research Ethics Committee, Dumfries and Galloway Health Board Local Research Ethics Committee, Forth Valley Health Board Local Research Ethics Committee, METC board of Leiden University Medical Center and the Clinical Research Ethics Committee of The Cork Teaching Hospitals], and all participants gave written informed consent. The protocol was consistent with the Declaration of Helsinki.

**Table 1:** Variables at baseline in the whole cohort not on warfarin and risk of definite VTE

	Cases (n = 48)	Non-cases (n = 5627)	Unadjusted Hazard ratio (95% CI)	P-value
***Conventional risk factors***				
Age (years)	74.9 (3.1)	75.3 (3.4)	0.83 (0.54, 1.28)	0.39
Body mass index (kg/m^2^)	28.5 (5.2)	26.8 (4.2)	1.09 (1.02, 1.15)	0.0075
Total cholesterol (mmol/L)	5.5 (1.1)	5.7 (0.9)	0.80 (0.57, 1.10)	0.17
Triglyceride (mmol/L)	1.5 (0.9)	1.5 (0.7)	0.86 (0.56, 1.34)	0.51
LDL (mmol/L)	3.6 (0.9)	3.8 (0.8)	0.78 (0.54, 1.12)	0.18
HDL (mmol/L)	1.3 (0.4)	1.3 (0.3)	1.04 (0.89, 1.21)	0.65
Systolic blood pressure (mmHg)	147.7 (21.9)	154.7 (21.8)	0.74 (0.57, 0.97)	0.027
Diastolic blood pressure (mmHg)	83.2 (12.4)	83.8 (11.4)	0.96 (0.74, 1.23)	0.73
Men	22 (45.8)	2694 (47.9)	0.94 (0.53, 1.66)	0.83
Current smoker	12 (25.0)	1519 (27.0)	0.93 (0.48, 1.79)	0.82
***History of vascular disease***				
History of hypertension	32 (66.7)	3493 (62.1)	1.22 (0.67, 2.22)	0.52
History of diabetes	5 (10.4)	603 (10.7)	1.00 (0.40, 2.53)	1.00
History of vascular disease	16 (33.3)	2446 (43.5)	0.66 (0.36, 1.20)	0.18
History of MI	7 (14.6)	722 (12.8)	1.19 (0.53, 2.65)	0.67
History of angina	13 (27.1)	1519 (27.0)	1.01 (0.54, 1.91)	0.97
History of CHD	15 (31.3)	1758 (31.2)	1.01 (0.55, 1.86)	0.97
History of claudication	2 (4.1)	371 (6.6)	0.63 (0.15, 2.58)	0.52
History of arterial surgery or amputation for vascular disease	2 (4.1)	312 (5.5)	0.75 (0.18, 3.10)	0.69
History of peripheral arterial disease	3 (6.3)	603 (10.7)	0.57 (0.18, 1.82)	0.34
History of stroke/TIA	4 (8.3)	615 (10.9)	0.75 (0.27, 2.10)	0.59
***Disability and cognition***				
IADL score	13.6 (1.1)	13.6 (1.0)	0.93 (0.72, 1.19)	0.55
Mini mental state exam (MMSE) score	27.6 (1.6)	28.0 (1.6)	0.84 (0.71, 0.99)	0.034
Barthel index score	19.7 (0.7)	19.8 (0.7)	0.93 (0.66, 1.30)	0.66
Years in education	15.0 (2.0)	15.1 (2.0)	0.98 (0.85, 1.13)	0.76
***Country of origin***				
Scotland	21 (43.8)	2466 (43.8)	4.26 (1.00, 18.21)	0.034
Ireland	25 (52.1)	2104 (37.4)	6.16 (1.46, 26.00)	
Netherlands	2 (4.2)	1057 (18.8)	Referent	
***Drug treatment***				
Pravastatin treatment	28 (58.3)	2795 (49.7)	1.42 (0.80, 2.52)	0.23
***Co-morbidities***				
Cancer during the study	11 (22.9)	415 (7.4)	4.58 (2.33, 9.01)	<0.0001
***Inflammation***				
IL-6* (pg/ml)	2.8 (2.1)	2.6 (1.9)	1.21 (0.80, 1.85)	0.37
sICAM-1* (ng/mL)	358 (1.41)	372 (1.39)	0.74 (0.31, 1.76)	0.50
CRP* (mg/L)	3.6 (3.1)	3.1 (3.1)	1.16 (0.90, 1.50)	0.25
Leptin* (ng/mL)	16.9 (2.4)	13.4 (2.4)	1.34 (0.97, 1.85)	0.077
***Lipids***				
Lp(a)* (mg/dL)	11.2 (2.8)	13.6 (3.5)	0.89 (0.71, 1.11)	0.30
Apo A1 (g/L)	1.3 (0.2)	1.3 (0.2)	0.92 (0.29, 2.97)	0.89
Apo B (g/L)	1.1 (0.2)	1.1 (0.2)	0.45 (0.12, 1.66)	0.23
***Biochemistry***				
Glucose* (mmol/L)	5.2 (1.2)	5.3 (1.2)	0.58 (0.13, 2.58)	0.47
Creatinine (umol/L)	104.2 (23.5)	101.1 (22.3)	1.01 (0.99, 1.02)	0.33
Urea*(mmol/L)	5.7 (1.4)	6.1 (1.3)	0.45 (0.16, 1.24)	0.12
ALT* (U/L)	22.3 (1.5)	21.3 (1.5)	1.32 (0.69, 2.54)	0.40
AST*(U/L)	24.3 (1.3)	23.5 (1.4)	1.38 (0.59, 3.21)	0.45
Creatine Kinase* (U/L)	84.0 (1.6)	82.6 (1.6)	1.06 (0.60, 1.86)	0.84
Free T4* (nmol/L)	17.6 (1.2)	16.3 (1.2)	1.77 (0.61, 5.15)	0.30
TSH* (mU/L)	1.6 (2.0)	1.8 (2.4)	0.89 (0.66, 1.20)	0.45
***Hematology***				
Hemoglobin (g/dL)	14.2 (1.2)	14.0 (1.2)	1.15 (0.92, 1.44)	0.22
Hematocrit (L/L)	0.4 (0.03)	0.4 (0.04)	1.22 (0.90, 1.65)	0.19

Mean and standard deviation (SD), *geometric mean (SD) calculated from the log transformed distribution or n (%) for categorical variables are shown. Hazard ratios and confidence intervals are for increases of 1 unit, except for age (increase of 5 years), SBP (increase of 20 mmHg), DBP (increase of 10 mmHg), hematocrit (increase of 0.04 L/L) and log free T4 (increase of 0.2 log unit).

**Table 2:** Plasma risk markers in nested case control study where subjects were not on warfarin

	Cases (n = 48)	Controls (n = 93)	Odds ratio (95% CI)	P-value
Factor VIII (iu/dL)	158.4 (42.0)	144.6 (38.2)	1.52 (1.01, 2.28)	0.044
Fibrinogen (g/L)	3.60 (0.61)	3.73 (0.79)	0.81 (0.54, 1.24)	0.34
PAI-1 (ng/mL)	58.5 (35.9)	58.8 (30.9)	0.97 (0.68, 1.38)	0.86
IGF-1 (ng/mL)	74.0 (19.2)	76.0 (24.3)	0.92 (0.63, 1.34)	0.66
Factor VII (iu/dL)	135.0 (32.8)	137.9 (32.5)	0.85 (0.56, 1.29)	0.45
Factor IX (iu/dL)	138.6 (30.0)	140.4 (26.8)	0.94 (0.66, 1.33)	0.72
APC ratio	3.83 (1.19)	3.87 (1.12)	0.96 (0.66, 1.40)	0.85

Means (SD) are shown. Unadjusted odds ratios for continuous variables with approximately 1 SD change (SD in control group) are shown.

### Baseline data

Baseline demographics, clinical history, activities of daily living (20-point Barthel index), instrumental activities of daily living (14-point IADL scale) and Mini Mental State Examination (MMSE) were collected as described previously[16].

### Baseline blood analyses

Baseline blood glucose, serum creatinine, urea, alanine aminotransferase (ALT), aspartate aminotransferase (AST), creatine kinase, free T4, thyroid stimulating hormone, hemoglobin, hematocrit, white blood cell count and platelet count were carried out by routine methods[17]. Baseline plasma lipids and lipoproteins were analyzed as described previously[18]. Baseline apolipoproteins AI and B were assayed by turbidimetric assays (Hitachi/Roche) as described previously[18]. Plasminogen Activator Inhibitor-1, interleukin-6, soluble Intracellular Adhesion Molecule-1 (sICAM-1) and Insulin-like Growth Factor-1 were assayed by commercial ELISA (Biopool, R&D). Baseline plasma leptin was measured by an 'in house' radioimmunoassay[19]. Baseline Factors VII, VIII & IX, fibrinogen and activated protein C (APC) ratio were assessed using commercial coagulation assays (MDA Coagulometer, Biomerieux, Basingstoke, UK). Baseline high sensitivity C-reactive protein (CRP)[20] and lipoprotein (a) (Lp(a))[21] were analyzed by automated particle-enhanced immunoturbimetric assay and latex agglutination assay respectively. For blood analyses listed in Table 1 data was already available in the PROSPER database for all participants. For the coagulation analytes and IGF-1, the analyses were carried out specifically for the purposes of the current study and were performed in cases and matched controls only due to limited resource.

### Statistical analysis

Data were analyzed using SAS vs9.1 (SAS Institute Inc, Cary NC). Where necessary continuous variables were transformed logarithmically to give a near-normal distribution of data for parametric analysis. Where data were available from the entire cohort, analyses were carried out using cases and non-cases (n = 5627) not using warfarin at baseline, including 2834 patients allocated to pravastatin and 2865 to placebo. The time to VTE was quantified by univariate hazard ratios and 95% confidence intervals calculated with Cox's proportional hazard model for each variable of interest. In the multivariate analyses, forward stepwise regression was undertaken, where all variables significant at the 5% level on univariate analysis were allowed to enter the model, to determine the subsets of variables that were independently associated with VTE. Where data were available only for the cases and controls (Factor VIII, fibrinogen, PAI-1, IGF-1, Factor VII, Factor IX, APC ratio), statistical analyses included calculation of the conditional logistic regression univariate odds ratio (which accounts for matching). For continuous variables, the odds ratio is that associated with 1 standard deviation difference (in the control group) in the variable of interest. A *post hoc *power calculation suggests that we had 60-97% power to detect an odds ratio of similar magnitude (0.4-0.6) to previously published observational studies [4-9].

## Results

### Pravastatin use and VTE

There were 28 cases of definite VTE in 2834 non-warfarinised patients in the pravastatin group and 20 cases in 2865 allocated to placebo (Additional file 1 Table S1). The unadjusted hazard ratio for VTE in the pravastatin versus placebo group was 1.42 (95% confidence interval 0.80, 2.52; p = 0.23) (Table 1). The results were similar after adjusting for cancer diagnosis in a multivariate analysis [1.36 (0.77, 2.42) p = 0.29] and when analysing only those participants without an incident cancer [1.51 (0.78, 2.90) p = 0.22]. An analysis was also carried out in the group combining a definite and a probable diagnosis of VTE where the unadjusted hazard ratio for VTE in the pravastatin versus placebo group was 1.20 (0.75, 1.90), p = 0.45.

### Clinical and plasma risk markers in entire PROSPER cohort

Baseline data for definite VTE cases and non-cases in the PROSPER cohort are shown in Table 1. Higher body mass index was associated with an increase in risk for VTE [1.09 (1.02, 1.15) p = 0.0075]. Systolic blood pressure (increase of 20 mmHg) was associated with a reduced risk of VTE [0.74 (0.57, 0.97) p = 0.027. Higher MMSE score was associated with a reduced risk of VTE [0.84 (0.71, 0.99) p = 0.034]. Subjects living in Scotland were 4.26 (1.00, 18.21) and subjects in Ireland were 6.16 (1.46, 26.00) times more likely to have a VTE compared to subjects living in the Netherlands (p = 0.034). In the stepwise model only BMI [1.09 (1.03, 1.16) p = 0.0053], systolic blood pressure (increase of 20 mmHg) [0.73 (0.56, 0.95) p = 0.021] and country [Scotland vs Netherlands 3.98 (0.93, 17.03) and Ireland vs Netherlands 5.62 (1.33, 23.76) p = 0.049] were independently predictive of VTE. Analysis using a combined group of definite and possible VTE gave similar results, apart from the association with systolic blood pressure which was not a significant predictor of combined VTE in both univariate and multivariate analysis.

### Plasma risk markers in nested case control study

Baseline data in the nested case (n = 48) control (n = 93) study for Factors VIII, fibrinogen, PAI-1, IGF-1, Factor VII, Factor IX and APC ratio are shown in Table 2. Plasma factor VIII levels were associated with an increased risk of definite VTE [1.52 (1.01, 2.28) p = 0.044]. None of these risk markers was associated with an increased risk of combined definite and probable VTE.

### Risk factor profile in cancer-associated VTE and non cancer-associated VTE

Cancer is a recognised risk factor for VTE. In the current study cases were divided into cancer-associated (n = 11) and non cancer-associated (n = 37) VTE. The hazard ratio for cancer as a predictor of VTE was 4.58 (2.33, 9.01), P < 0.001 when comparing cases with non-cases (Table 1). Including cancer as a variable in the stepwise multivariate model had no impact on the associations we observed. There were more new cases of cancer in the pravastatin group than in the placebo group [15] (Additional file 1 Table S1) and since the etiology of VTE might differ between those with a diagnosis of cancer and those without we explored the possibility that baseline risk factor profile differed between cases who had cancer and cases who did not have cancer. We classified cancer-associated VTE as those individuals who had a VTE subsequent to their cancer diagnosis (n = 5) and those who had a VTE prior to their cancer diagnosis (n = 6), total 22.9%. There were no differences in risk factor profile between those who had the VTE before and those who had a VTE after their cancer diagnosis.

## Discussion

This study showed with data from a randomized controlled trial a lack of a protective effect of pravastatin on VTE incidence in the elderly [1.42 (0.80, 2.52) p = 0.23]. The recently published data from the JUPITER study[11] indicated that rosuvastatin significantly reduced the occurrence of symptomatic VTE [0.57 (0.37, 0.86) p = 0.007] in middle-aged subjects with low LDL cholesterol and raised C-reactive protein levels. The PROSPER study had 48 VTE events (from 18,363 person-years of follow-up in those not on warfarin), fewer than the 94 events seen in JUPITER (17,802 participants with median follow up of 1.9 years). However incidence rates were similar: 0.26% for PROSPER and 0.28% for JUPITER. In PROSPER new cancer diagnoses were more frequent in the pravastatin treated group than in the placebo treated group[15]. However the lack of effect of pravastatin on VTE incidence was also observed when only considering participants with no diagnosis of cancer. Interestingly a very large, unselected population-based cohort study (n = 129,288) looking at statin use and a number of health outcomes [22] found a similar lack of effect of statins on VTE incidence to that observed in PROSPER.

The differing results in the PROSPER and JUPITER studies may be explained by the different characteristics of the populations. PROSPER was an elderly, high risk population. JUPITER was carried out in a younger, initially healthy population although they did note a similar, but not statistically significant, benefit in a high risk subgroup of elderly participants ≥ 70 years of age. In PROSPER, LDL cholesterol levels were approximately 1.0 mmol/L higher and CRP levels approximately 1.0 mg/L lower than those observed in the JUPITER study. It is possible that more VTE events in JUPITER had an underlying etiology involving pro-inflammatory pathways [23] that may be susceptible to statin intervention. Notably inflammation markers, and in particular CRP, were not independently linked to VTE risk in PROSPER. Since our study involved the use of pravastatin we cannot rule out the possibility that the apparent lack of efficacy in VTE prevention is confined to pravastatin alone. However both pravastatin and rosuvastatin which was used in JUPITER are water-soluble statins and thus differences in tissue distribution are unlikely to explain the difference between the effects of these drugs on VTE events. Individual data meta-analyses of VTE risk in randomized placebo controlled trials with statins would be useful to address this issue and the low power of single trials. Nevertheless, our results go against a class effect of statins in VTE which is somewhat surprising since all statins lessen cardiovascular disease risk including in the elderly.

BMI, lower systolic blood pressure and country of origin were the only significant independent predictors of VTE in PROSPER. BMI was previously observed to be a risk factor for VTE in the prospective Copenhagen City Heart Study [24], Physicians' Health Study[25] and LITE study[26] where the subjects were predominately aged <70 years. BMI appears to be more strongly associated with VTE than with either stroke or coronary heart disease[25]. In our elderly population the magnitude of risk associated with BMI appears less than that observed in younger populations. It is possible that the association between BMI and VTE is attenuated by age. Many mechanisms by which obesity may increase the risk of VTE have been proposed, including promotion of inflammation by adipokines, increased coagulation activity, decreased fibrinolysis, increased oxidative stress, metabolic disturbances and endothelial dysfunction[27]. We found that low systolic blood pressure was an independent risk factor for incident VTE. This contrasts with prospective studies of VTE in younger adults, in whom a positive association has been observed with high blood pressure [24]. However it is possible that there is a change in the relationship of blood pressure with risk of VTE with advancing age. In support of this blood pressure levels in very elderly people have an apparently paradoxical inverse relationship with risk of other adverse clinical events including total mortality. Low blood pressure in elderly people is likely to be associated with more severe underlying covert disease including coronary heart disease; often these underlying diseases are unrecognized because there may be no classical symptoms [28]. It is likely that low blood pressure in older people is a risk marker for VTE, rather than directly causative. The lower incidence of definite VTE in the Netherlands (0.19%) compared to Scotland (0.84%) and Ireland (1.17%) is likely to reflect a healthier cohort in this country; in the Netherlands there was lower incidence of most recorded adverse outcomes, including ischemic vascular events, compared to the other 2 countries. Baseline BMI was not different between randomised subjects from each country [16]. Our finding that coagulation Factor VIII was associated with risk of definite DVT is consistent with reports from several other cross-sectional studies, and from two prospective studies (reviewed in [29]).

There were limitations to our study. It is a *post hoc *analysis on a subgroup from the PROSPER study. The subjects were from a selected population with either evidence of pre-existing vascular disease or at least one major risk factor for vascular disease. Thus our results can only be applied to such high risk individuals. However this is the very subgroup of older people who are likely to be considered for statin therapy. The number of VTE is small and we may not be able to detect a small effect of pravastatin treatment. The incidence of definite VTE in the current study of 0.26% per annum is less than that predicted for the very old (1% per annum[1]) but equivalent to that observed in JUPITER (0.28% per annum). This may have been due to a higher proportion of relatively healthy and active individuals volunteering to take part in a randomized controlled trial, the use of cancer at baseline as an exclusion criterion and insufficient recorded data to identify all cases. The analysis presented here used definite VTE cases only however analysis using a combination of definite and probable VTE gave similar results. The PROSPER database did not have information on transient or precipitating risk factors, other than cancer, that may have preceded a VTE, such as immobilization or previous general surgery.

## Conclusions

In conclusion we found that pravastatin does not prevent VTE in elderly people at risk of vascular disease. Blood markers of haemostasis and inflammation are not strongly predictive of VTE in the elderly however BMI, country and lower systolic blood pressure are independently associated with VTE risk.

## Abbreviations

ALT: alanine aminotransferase; apo: apolipoprotein; APC: activated protein C; AST: aspartate aminotransferase; BMI: body mass index; CHD: coronary heart disease; CRP: C reactive protein; DBP: diastolic blood pressure; HDL: high density lipoprotein; IADL: instrumental activities of daily living score; IGF-1: insulin-like growth factor 1; IL-6: interleukin 6; LDL: low density lipoprotein; Lp(a): lipoprotein (a); MI: myocardial infarction; MMSE: mini mental state exam; PAI-I: plasminogen activator inhibitor 1; PROSPER: Prospective Study of Pravastatin in the Elderly at Risk; SBP: systolic blood pressure; sICAM-1: soluble intercellular adhesion molecule; SD: standard deviation; T4: thyroxine; TIA: transient ischemic attack; TSH: thyroid stimulating hormone; VTE: venous thromboembolism.

## Competing interests

The authors declare that they have no competing interests.

## Authors' contributions

DJF, IF, AG, IAG, GDOL and DJS were involved in the initial conception and design of the study. AJMdC, IF, AG, JWJ, NS and DJS were investigators in the PROSPER study. Data collection was carried out by EAB, ER, EST, MF and PES. IF and MR were responsible for data retrieval from the PROSPER database and statistical analysis. DJF, AG, IAG, GDOL, MR and DJS were responsible for the main data interpretation and analysis. All authors contributed to the writing of this manuscript. All authors have read and approved the final manuscript.

## Pre-publication history

The pre-publication history for this paper can be accessed here:

http://www.biomedcentral.com/1471-2318/11/8/prepub

## Supplementary Material

Additional file 1 **Table S1**. Characteristics at baseline by treatment group in PROSPER participants not on warfarin Click here for file
