# Supplementary material for: Incident venous thromboembolic events in the Prospective Study of Pravastatin in the Elderly at Risk (PROSPER)
Source: BMC Geriatr. 2011 Feb 22;11:8. doi: 10.1186/1471-2318-11-8 (PMC3053238; doi:10.1186/1471-2318-11-8)
Supplement: Additional file 1 — Table S1. Characteristics at baseline by treatment group in PROSPER participants not on warfarin [file 1471-2318-11-8-S1.DOC]

## Additional file 1 Table S1 - Characteristics at baseline by treatment group in PROSPER participants not on warfarin

Mean and standard deviation (SD), *geometric mean (SD) calculated from the log transformed distribution or n (%) for categorical variables are shown.

|  | **Placebo**  **(n=2865)** | **Pravastatin (n=2834)** |
| --- | --- | --- |
| ***VTE*** | | |
| Definite | 20 (0.70) | 28 (0.99) |
| Definite and probable | 33 (1.15) | 39 (1.38) |
| ***Cancer*** | | |
| New cases in PROSPER | 196 (6.8) | 235 (8.3) |
| ***Conventional risk factors*** | | |
| Age (years) | 75.3 (3.4) | 75.4 (3.3) |
| Body mass index (kg/m^2^) | 26.8 (4.3) | 26.8 (4.1) |
| Total cholesterol (mmol/L) | 5.7 (0.9) | 5.7 (0.9) |
| Triglyceride (mmol/L) | 1.5 (0.7) | 1.6 (0.7) |
| LDL (mmol/L) | 3.8 (0.8) | 3.8 (0.8) |
| HDL (mmol/L) | 1.3 (0.3) | 1.3 (0.4) |
| Systolic blood pressure (mmHg) | 154.6 (21.7) | 154.7 (21.9) |
| Diastolic blood pressure (mmHg) | 83.9 (11.7) | 83.6 (11.2) |
| Men | 1376 (48.0) | 1352 (47.7) |
| Current smoker | 794 (27.7) | 743 (26.2) |
| *History of vascular disease* | |  |
| History of hypertension | 1768 (61.7) | 1771 (62.5) |
| History of diabetes | 315 (11.0) | 296 (10.4) |
| History of vascular disease | 1221 (42.6) | 1253 (44.2) |
| History of MI | 379 (13.2) | 354 (12.5) |
| History of angina | 752 (26.2) | 789 (27.8) |
| History of CHD | 877 (30.6) | 906 (32.0) |
| History of claudication | 187 (6.5) | 190 (6.7) |
| History of arterial surgery or amputation for vascular disease | 146 (5.1) | 168 (5.9) |
| History of peripheral arterial disease | 298 (10.4) | 312 (11.0) |
| History of stroke/TIA | 313 (10.9) | 308 (10.9) |
| ***Disability and cognition*** | |  |
| IADL score | 13.6 (1.0) | 13.6 (1.0) |
| Mini mental state exam (MMSE) score | 28.0 (1.6) | 28.0 (1.5) |
| Barthel index score | 19.8 (0.7) | 19.8 (0.8) |
| Years in education | 15.1 (2.0) | 15.2 (2.1) |
| *Country of origin* |  |  |
| Scotland | 1249 (43.6) | 1247 (44.0) |
| Ireland | 1083 (37.8) | 1058 (37.3) |
| Netherlands | 533 (18.6) | 529 (18.7) |
| *Inflammation* |  |  |
| IL-6* (pg/ml) | 2.6 (1.9) | 2.7 (1.9) |
| sICAM-1* (ng/mL) | 370 (1.40) | 373 (1.38) |
| CRP* (mg/L) | 3.1 (3.0) | 3.1 (3.1) |
| Leptin* (ng/mL) | 13.4 (2.4) | 13.4 (2.4) |
| *Lipids* |  |  |
| Lp(a)* (mg/dL) | 13.5 (3.5) | 13.6 (3.5) |
| Apo A1 (g/L) | 1.3 (0.2) | 1.3 (0.2) |
| Apo B (g/L) | 1.1 (0.2) | 1.2 (0.2) |
| *Biochemistry* |  |  |
| Glucose* (mmol/L) | 5.3 (1.2) | 5.3 (1.2) |
| Creatinine (umol/L) | 101.0 (22.3) | 101.3 (22.4) |
| ­Urea*(mmol/L) | 6.1 (1.3) | 6.0 (1.3) |
| ALT* (U/L) | 21.4 (1.5) | 21.1 (1.5) |
| AST*(U/L) | 23.6 (1.4) | 23.5 (1.4) |
| Creatine Kinase* (U/L) | 82.7 (1.6) | 82.5 (1.6) |
| Free T4* (nmol/L) | 16.2 (1.2) | 16.4 (1.2) |
| TSH* (mU/L) | 1.8 (2.4) | 1.7 (2.3) |
| *Hematology* |  |  |
| Hemoglobin (g/dL) | 14.1 (1.2) | 14.0 (1.2) |
| Hematocrit (L/L) | 0.4 (0.04) | 0.4 (0.04) |
